# First record of non‐flying mammalian contributors to pollination in a tropical montane forest in Asia

**DOI:** 10.1002/ece3.8361

**Published:** 2021-12-03

**Authors:** Shun Kobayashi, Somsak Panha, Teerapong Seesamut, Nattawadee Nantarat, Natdanai Likhitrakarn, Tetsuo Denda, Masako Izawa

**Affiliations:** ^1^ Faculty of Science University of the Ryukyus Nishihara Japan; ^2^ Department of Biology Faculty of Science Chulalongkorn University Bangkok Thailand; ^3^ Academy of Science The Royal Society of Thailand Bangkok Thailand; ^4^ Department of Biology Faculty of Science Chiang Mai University Chiang Mai Thailand; ^5^ Faculty of Agricultural Production Maejo University Chiang Mai Thailand; ^6^ Present address: Kitakyushu Museum of Natural History and Human History Kitakyushu Japan

**Keywords:** montane forest, *Mucuna thailandica*, non‐flying mammal, pollination, tropical Asia

## Abstract

This study aims to identify the flower visitors of *Mucuna thailandica* (Fabaceae), endemic plant species in montane forests in Thailand, to determine their potential pollinators. The genus *Mucuna* produces papilionaceous flowers and has an explosive flower‐opening step. Explosive opening rapidly exposes stamens and pistil from keel petals and releases pollen. The flower of this species depends completely on animals to perform this step, essential for pollination success. Using a camera trap survey, we revealed that non‐flying mammals, such as squirrels (*Callosciurus* sp.) and masked palm civets (*Paguma larvata*), opened flowers explosively. Thus, these mammals contribute to the pollination of *M. thailandica*. This is the first report of non‐flying mammals contributing to pollination in montane forests in tropical Asia.

## Graphical abstract

This study aimed to reveal the contributors to pollination of *Mucuna thailandica* (Fabaceae), which is a woody vine and is distributed in the montane forests in Thailand. A camera trap survey demonstrated that non‐flying mammals, such as squirrels (*Callosciurus* sp.) and masked palm civets (*Paguma larvata*) contribute to pollination of *M. thailandica*.

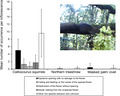

## 1 INTRODUCTION

Bird‐ and mammal‐pollinated plants are recorded from 28 orders and 67 families in the world (Fleming & Kress, 2013). Most mammal‐pollinated plants are pollinated by bats: however, non‐flying mammals have also been recorded to pollinate some plant species (Carthew & Goldingay, 1998; Willmer, 2011). These plants are distributed across different regions, including semi‐arid regions, shrublands, and montane grasslands in Africa (Hobbhahn et al., 2017; Kleizen et al., 2008; Payne et al., 2019; Steenhuisen et al., 2015; Wester et al., 2019), cloud forests, rainforests, and alpine dry shrubland and grasslands in Central and South America (Amorim et al., 2020; Cárdenas et al., 2017; Cocucci & Sersic, 1998; Dellinger et al., 2019; Lumer, 1980), and heathland, dry woodland, and rain forests in Australia (Carpenter, 1978; Carthew, 1993; Goldingay et al., 1991; Quin et al., 1996; Wooller & Wooller, 2003). Such wide distribution of mammal‐pollinated plants suggests that non‐flying mammal‐pollinated plants could exist in various habitats across these regions. However, only a few species of non‐flying mammal‐pollinated plants have been recorded in tropical Asia (Carthew & Goldingay, 1998; Willmer, 2011), and these records are limited to lowland evergreen forests (Ganesh & Devy, 2000, 2006; Kobayashi, Denda, et al., 2019; Yumoto et al., 2000).

In particular, non‐flying mammal‐pollinated plants have not been recorded in tropical Asian montane forests. In the aseasonal tropics in Asia, the difference in altitude between lowland and montane habitats ranges from 750 to 1300 m a.s.l. (Ashton, 2018). Montane areas can be classified into lower montane, upper montane, and subalpine areas where the tree line is above 3900–4000 m a.s.l. (Corlett, 2019). Bees are the most important pollinators in areas up to 1500 m a.s.l. (reviewed by Corlett, 2004).

**FIGURE 1:**
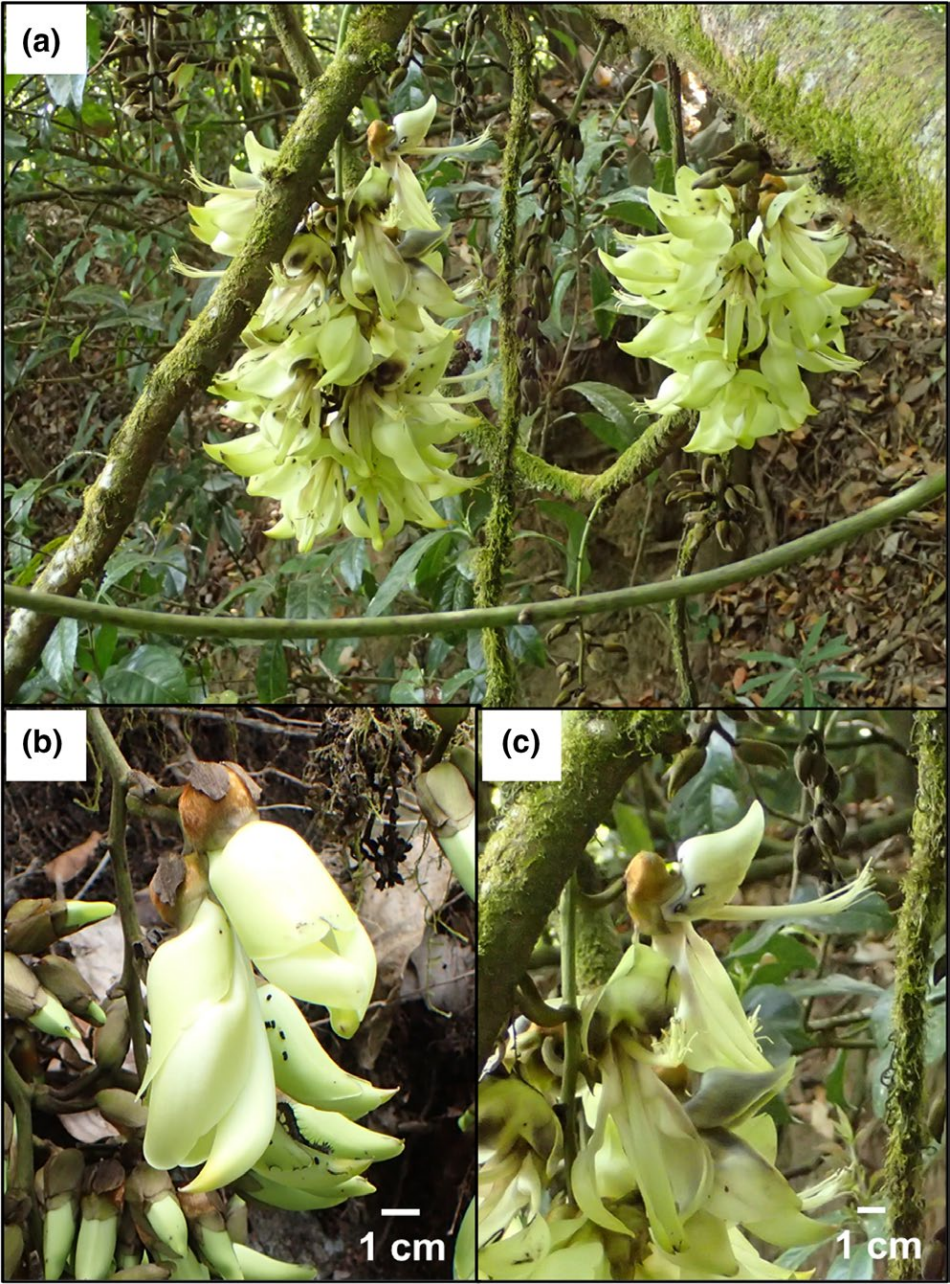
Inflorescences and flowers of *Mucuna thailandica*. (a) Inflorescences; (b) flowers before they were explosively opened, (c) flowers after they were explosively opened

One characteristic of mammal‐pollinated plants is that they tend to have relatively large flowers or inflorescences compared to entomophilous (insect‐pollinated) species (Fleming & Kress, 2013). Such plants can be found in lowlands and montane forests in tropical Asia. One of these species is *Mucuna thailandica* Niyomdham & Wilmot‐Dear. *Mucuna thailandica*, an evergreen woody vine distributed in an altitude range of 1000–2400 m a.s.l. and endemic to Doi Inthanon, the highest mountain in Thailand (Wilmot‐Dear, 2008). This species produces racemes with 18–45 pale green flowers that are more than 8 cm in length (Figure 1).

*Mucuna thailandica* has a typical papilionaceous (butterfly‐like) flower, consisting of a banner petal, a pair of wing petals, and a pair of keel petals that cover the stamens and pistils. Keel petal‐opening is essential for pollination in this genus, but they do not open by themselves (Agostini et al., 2006; Kobayashi, Denda, et al., 2019; Kobayashi et al., 2021). Rather, when an animal pushes the banner petal and presses the wing petals downward, the keel petals are opened and the stamens and pistil are exposed, upon which the pollen grains splash. This essential step is called “explosive opening.” Once a flower explosively opens, the stamens and pistil remain exposed. *Mucuna macrocarpa* and *M. birdwoodiana*, two related species, cannot produce seeds without an explosive opening step (Kobayashi, Denda, et al., 2019). In addition, the flower opening strength, as measured using a digital force gauge, was found to be 16–409 times higher for the explosive opening of *M. macrocarpa*, which is pollinated by squirrels (*Callosciurus* spp.), flying foxes (*Pteropus dasymallus*), and macaques (*Macaca fuscata*), than for bee‐pollinated plants (Kobayashi et al., 2018). These characteristics suggest that *M. thailandica* can be pollinated by mammals.

In this study, we identified flower visitors to the large flower plant *M. thailandica* in a montane forest in tropical Asia and recorded their flower‐visiting behavior to determine the potential contribution of non‐flying mammals to its pollination.

## 2 MATERIALS AND METHODS

The study was conducted at the Kew Mae Pan Nature Trail in Doi Inthanon National Park, Thailand (18°33′20.63″N, 98°28′55.42″E, 2100 m a.s.l.). *Mucuna thailandica* has only been recorded in ten collections from Doi Inthanon and is thus very rare (Wilmot‐Dear, 2008). Only one individual was selected because of the suitability for the camera trap survey. The studied plant was located near the office of the bushwalk tour. Although tourists were allowed to bushwalk near the study site, tourism decreased drastically at the end of the flowering season due to the COVID‐19 pandemic, reducing the likely amount of disturbance. An automatic video camera trap (Ltl‐6210; Shenzhen Ltl Acorn Electronics Co., Ltd., China) with a night vision recording function using an infrared lamp was set up to document seven inflorescences from January 20 to July 9, 2020. These inflorescences were located approximately 3.2 m above ground. The distance between the camera and inflorescences was approximately 1 m. The selected settings of the camera were 30 s video length, without intervals, and a normal sensor level. We monitored the plant from the bud stage to the end of the flowering phase. Flower‐visiting behaviors were categorized based on Kobayashi et al. (2021) as: “explosive opening with no damage to the flower,” “visiting and feeding on the nectar of the opened flower,” “destruction of the flower without opening,” “nectar robbing from the unopened flower,” and “other non‐specific behaviors and unknown.” Fruit availability on monitored inflorescences was checked when the camera trap was collected on August 29, 2020.

## 3 RESULTS AND DISCUSSION

The first flower visitor was recorded on February 13, and all flowers dropped by March 31. In total, 173 shots were recorded. Animals were captured in 90 (52%) video shots, while the rest did not include animals. Tree squirrels (*Callosciurus* spp.), northern tree shrews (*Tupaia belangeri*), and masked palm civets (*Paguma larvata*) were recorded as flower visitors. Three *Callosciurus* species (*C. caniceps*, *C. erythraeus*, and *C*. *finlaysonii*) inhabit this area (Duckworth, 2016, 2017; Duckworth et al., 2017), but the flower‐visiting squirrel could not be identified because we used camera traps with a night vision recording function using an infrared lamp and thus could not differentiate the color or detailed characteristics in some cases. *Callosciurus* squirrels visited all seven monitored inflorescences; on the other hand, the northern tree shrew and masked palm civet visited only two inflorescences. Giant squirrel (*Ratufa indica*), Asian red‐cheeked squirrel (*Dremomys rufigenis*), bat sp., passerine birds, and sunbirds were also recorded in shots, but they did not visit the flowers.

**FIGURE 2:**
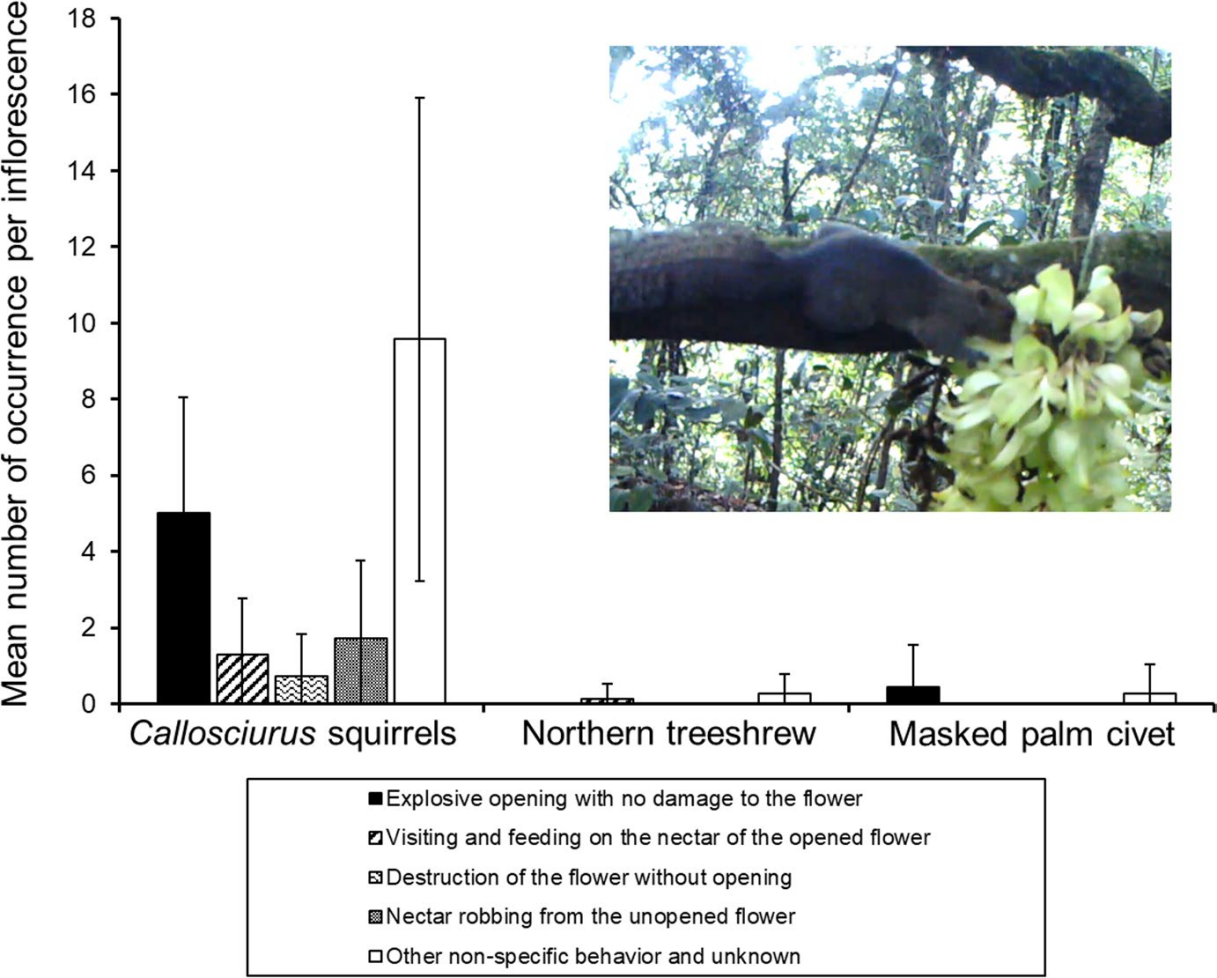
Flower visitors and their behaviors toward flowers. Photograph in the graph shows a *Callosciurus* squirrel holding a flower for explosive opening

Among these behaviors, “explosive opening with no damage to the flower” directly contributed to pollination success and was observed during visits by *Callosciurus* squirrels and masked palm civets. When the *Callosciurus* squirrels visited flowers, they frequently explosively opened flowers with no damage (5.0 ± 3.1 flowers per inflorescence; Figure 2). They grabbed a flower and then turned it upside down. After that, they opened the flower by holding it with their hands and pushing up the banner petal with their snout (Video S1). After the flower was opened, they fed on nectar stored inside the calyx. This was similar to the flower opening behavior previously reported for the squirrels *C. finlaysonii* and *C. caniceps* on *M. macrocarpa* (Kobayashi, Denda, et al., 2019). When the flowers were opened upside down, the stamens and pistils attached to the throat and head of the squirrels. However, when they opened flowers in an upright manner, the stigma did not attach to visitors. This behavior was often observed in immature flowers.

The masked palm civet opened only three flowers (0.4 ± 1.1 flowers per inflorescence; Figure 2). The flower opening behavior was almost the same as that of squirrels: stamens and pistils were attached to the throat of the masked palm civet (Video 2). This explosive opening behavior of the masked palm civet was similar to that previously reported for *M. birdwoodiana* flowers (Kobayashi et al., 2019). Pollen grains may attach to the throat, although the details are not clear because of the recording mode of the camera trap.

Flower‐opening animals are considered pollinators because the pollen and stigma attach under their jaws (Kobayashi, Denda, et al., 2019; Kobayashi et al., 2021). This attachment position may be related to the behavior of the visitor in opening the flower. Effective pollinators in previous studies including flying foxes turned flowers upside down and then opened flowers (Kobayashi et al., 2017; Kobayashi, Denda, et al., 2019; Toyama et al., 2012). In addition, explosive opening is essential for fruit set in other *Mucuna* species, such as *M. macrocarpa* and *M. birdwoodiana* (Kobayashi, Denda, et al., 2019). Similar results were observed in this study; one fruit was produced from a flower that had been opened by *Callosciurus* squirrel. Thus, *Callosciurus* squirrels and masked palm civets, which opened flowers, contribute to the pollination success of *M. thailandica*, and *Callosciurus* squirrels were considered the main pollinators of this plant.

Plants with greenish petals are usually pollinated by bats (Lau, 2004; van der Pijl, 1941) and small non‐flying mammals, for example, the case of mice and elephant‐shrews pollinating plants with green petals in South Africa (Wester, 2011; Wester et al., 2019). The plants in *Mucuna* are not always pollinated by bats, although bats inhabit the study site (Kobayashi, Gale, et al., 2019). In Doi Inthanon, at least one fruit bat species has been recorded (Thai National Parks, 2021), but no fruit bats visited *M. thailandica* flowers. Because the number of bat species is lower at higher elevations (McCain, 2011), relatively large flowers in such areas may be more likely to be pollinated by non‐flying mammals.

Information on plant–mammal interactions in montane forests, especially pollination‐mediated relationships, is still lacking in tropical Asia (Corlett, 2004; Funamoto, 2019), although it has been reported that non‐flying mammals contribute to pollination in other montane regions (Dellinger et al., 2019; Lumer, 1980). The present study showed that non‐flying mammals may play an important role as pollinators in tropical montane forests, although the sample size was insufficient. Non‐flying mammals are considered to be less important (Carthew & Goldingay, 1998; Willmer, 2011). Conversely, recent observations (Kobayashi, Denda, et al., 2019; Kobayashi et al., 2021) suggest that non‐flying mammals play an important role as pollinators in Asia and other regions.

However, one caveat is that mammals with a high wariness probably did not visit the flowers as the study site was located near the office of the bushwalk tour, and human activity was relatively high. This suggests that pollinators and their actual contribution to pollination in this plant may differ in the natural environment, and thus, further observations in an environment with less human presence would be beneficial to support our conclusions. Overall, more studies on plant–animal interactions are needed in montane forests in tropical Asia.

## CONFLICT OF INTEREST

The authors declare that they have no conflicts of interest.

## AUTHOR CONTRIBUTIONS

**Shun Kobayashi:** Conceptualization (equal); Data curation (equal); Funding acquisition (equal); Methodology (equal); Writing‐original draft (lead); Writing‐review & editing (equal). **Somsak Panha:** Conceptualization (equal); Funding acquisition (equal); Writing‐original draft (equal); Writing‐review & editing (equal). **Teerapong Seesamut:** Data curation (equal); Writing‐original draft (equal). **Nattawadee Nantarat:** Data curation (equal); Writing‐original draft (equal). **Natdainai Likhitrakarn:** Data curation (equal); Writing‐original draft (equal). **Tetsuo Denda:** Conceptualization (equal); Writing‐original draft (equal); Writing‐review & editing (equal). **Masako Izawa:** Conceptualization (equal); Methodology (supporting); Writing‐original draft (equal); Writing‐review & editing (equal).

## Supporting information

Video S1

Video S2

## Data Availability

The raw data used in this study are publicly available in the Dryad: https://orcid.org/0000‐0003‐3167‐3358.
